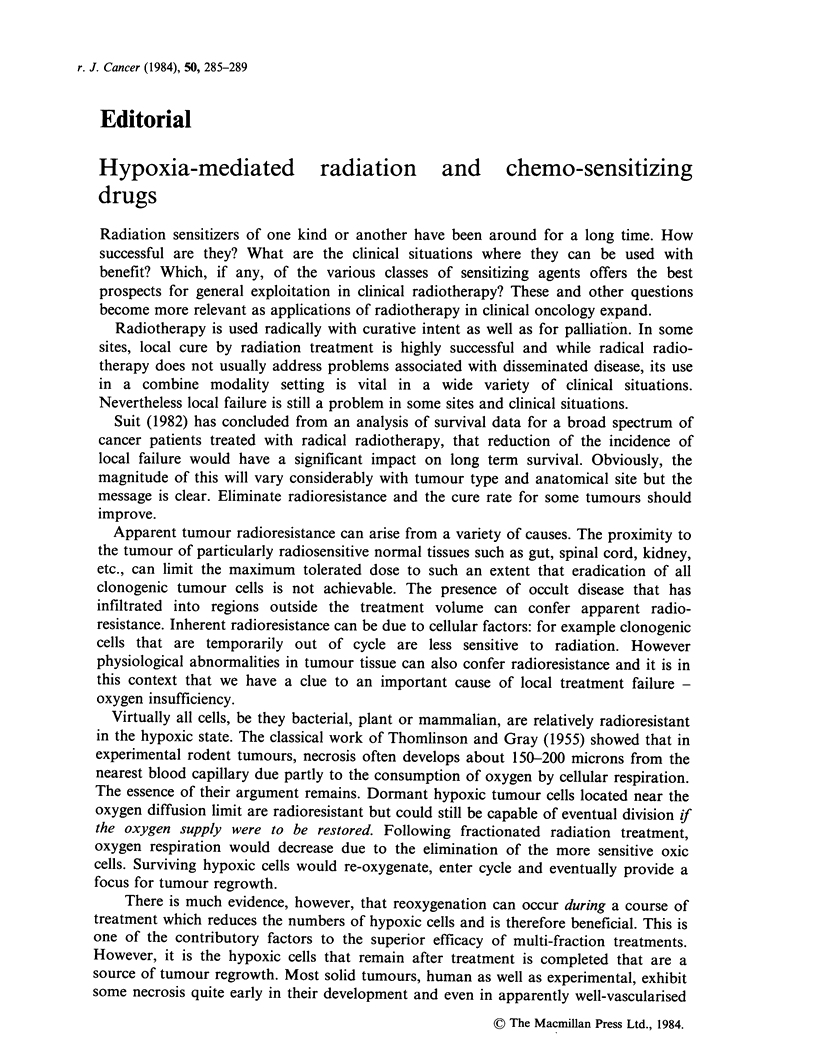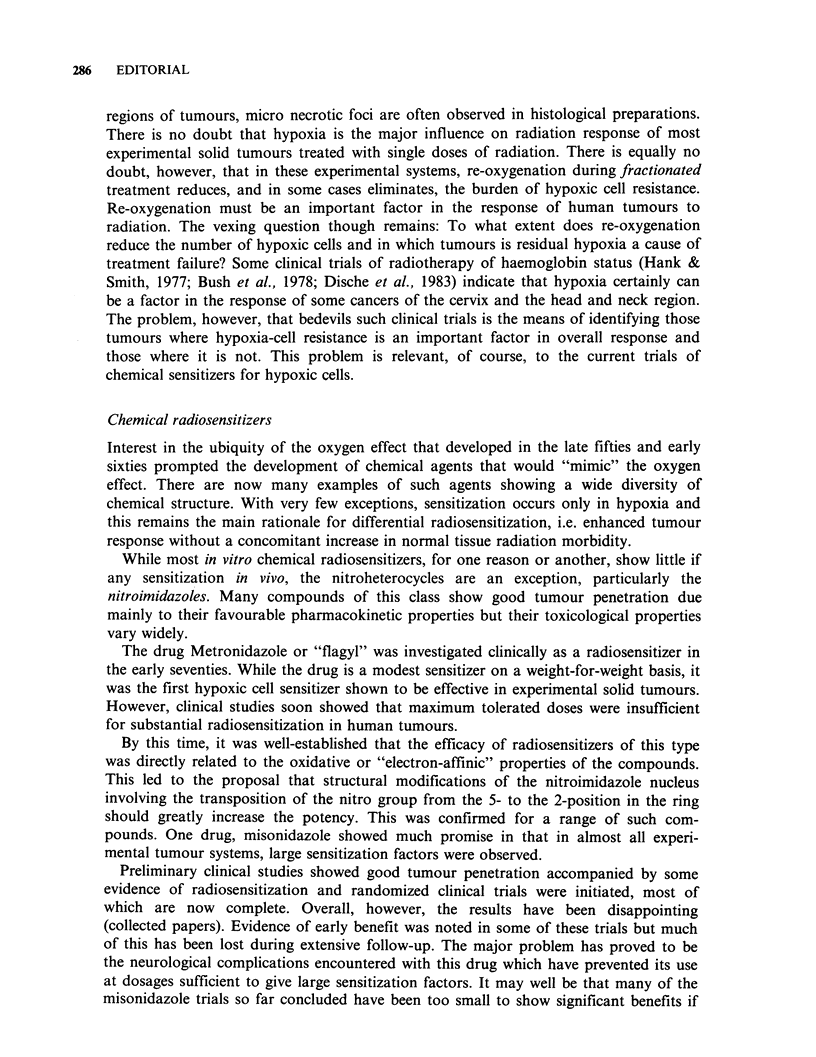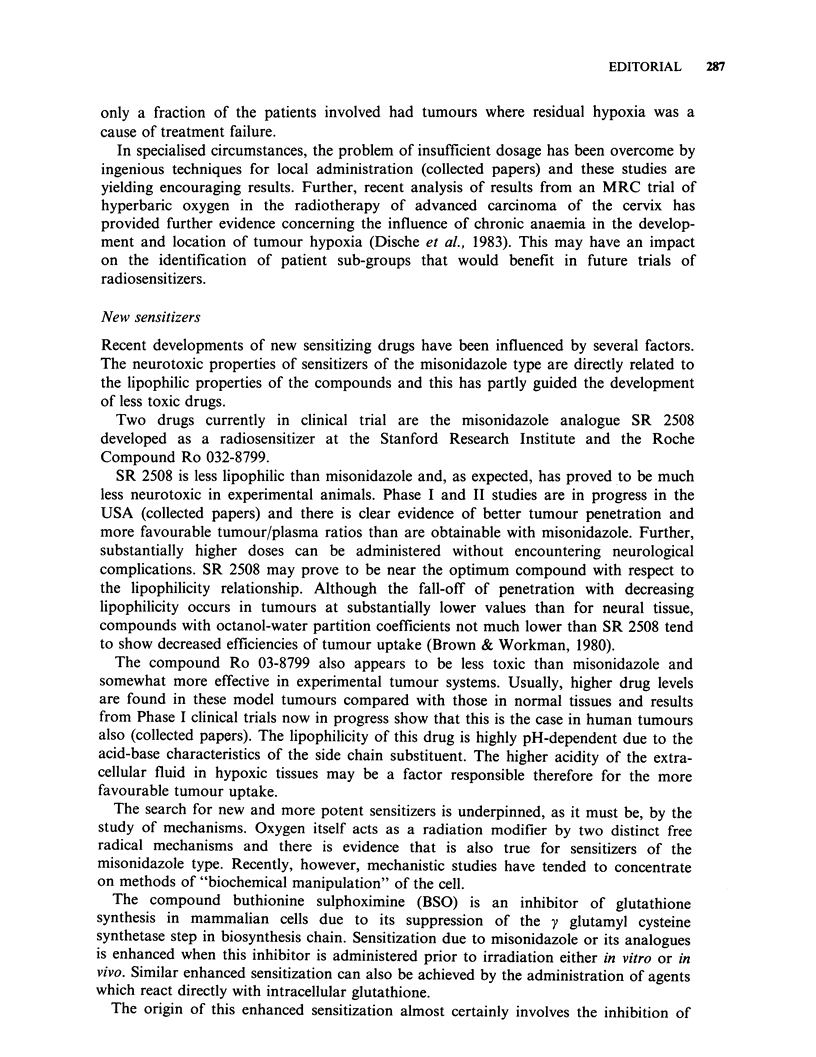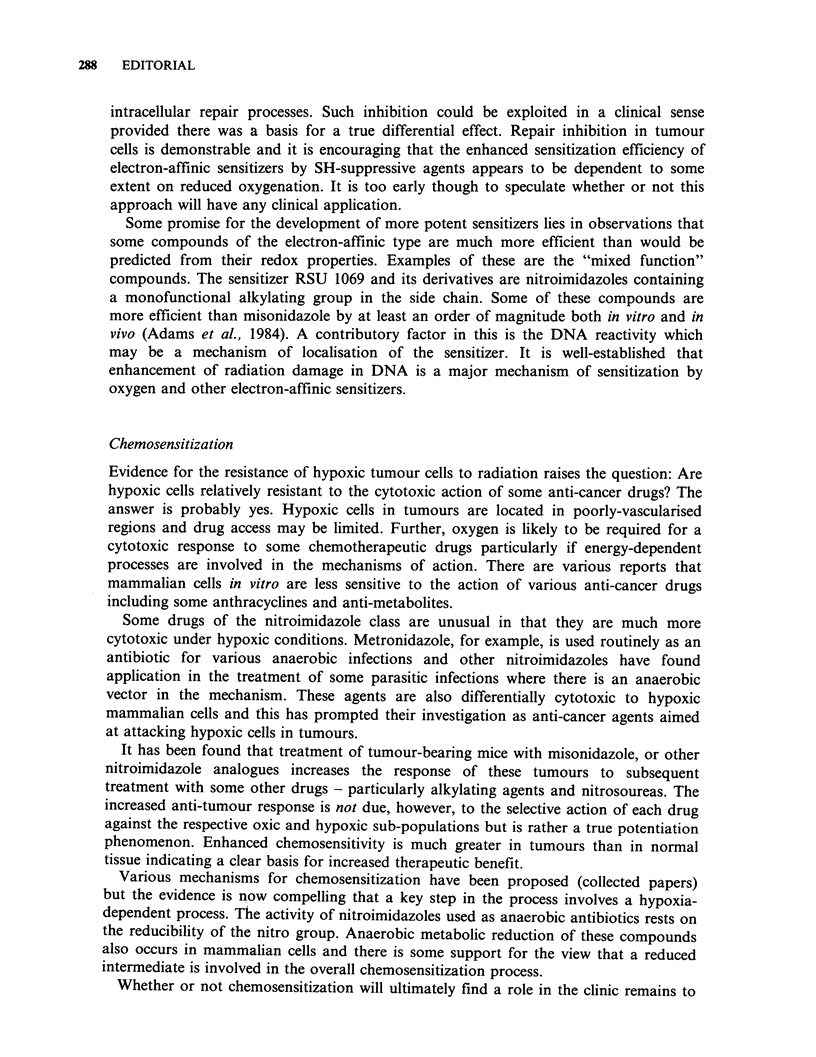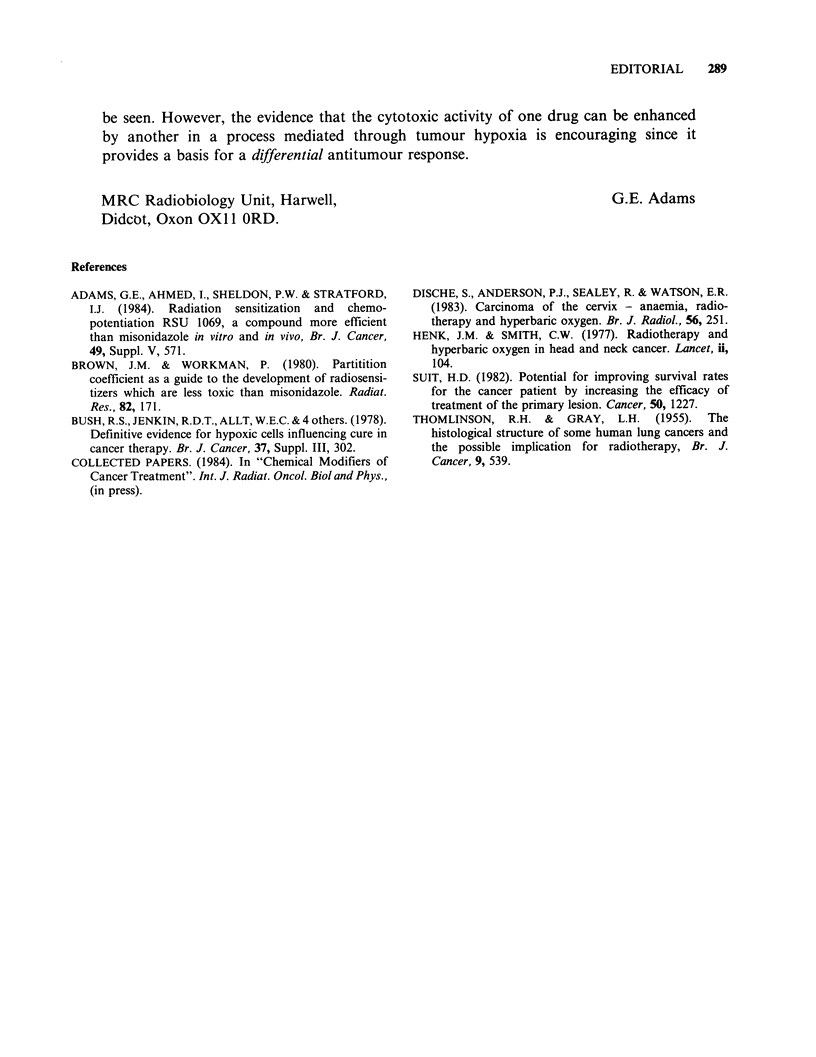# Hypoxia-mediated radiation and chemo-sensitizing drugs.

**DOI:** 10.1038/bjc.1984.175

**Published:** 1984-09

**Authors:** G. E. Adams


r. J. Cancer (1984), **50**, 285–289

### Editorial

### Hypoxia-mediated radiation and chemo-sensitizing drugs

Radiation sensitizers of one kind or another have been around for a long time. How successful are they? What are the clinical situations where they can be used with benefit? Which, if any, of the various classes of sensitizing agents offers the best prospects for general exploitation in clinical radiotherapy? These and other questions become more relevant as applications of radiotherapy in clinical oncology expand.

Radiotherapy is used radically with curative intent as well as for palliation. In some sites, local cure by radiation treatment is highly successful and while radical radiotherapy does not usually address problems associated with disseminated disease, its use in a combine modality setting is vital in a wide variety of clinical situations. Nevertheless local failure is still a problem in some sites and clinical situations.

Suit (1982) has concluded from an analysis of survival data for a broad spectrum of cancer patients treated with radical radiotherapy, that reduction of the incidence of local failure would have a significant impact on long term survival. Obviously, the magnitude of this will vary considerably with tumour type and anatomical site but the message is clear. Eliminate radioresistance and the cure rate for some tumours should improve.

Apparent tumour radioresistance can arise from a variety of causes. The proximity to the tumour of particularly radiosensitive normal tissues such as gut, spinal cord, kidney, etc., can limit the maximum tolerated dose to such an extent that eradication of all clonogenic tumour cells is not achievable. The presence of occult disease that has infiltrated into regions outside the treatment volume can confer apparent radioresistance. Inherent radioresistance can be due to cellular factors: for example clonogenic cells that are temporarily out of cycle are less sensitive to radiation. However physiological abnormalities in tumour tissue can also confer radioresistance and it is in this context that we have a clue to an important cause of local treatment failure – oxygen insufficiency.

Virtually all cells, be they bacterial, plant or mammalian, are relatively radioresistant in the hypoxic state. The classical work of Thomlinson and Gray (1955) showed that in experimental rodent tumours, necrosis often develops about 150–200 microns from the nearest blood capillary due partly to the consumption of oxygen by cellular respiration. The essence of their argument remains. Dormant hypoxic tumour cells located near the oxygen diffusion limit are radioresistant but could still be capable of eventual division *if the oxygen supply were to be restored.* Following fractionated radiation treatment, oxygen respiration would decrease due to the elimination of the more sensitive oxic cells. Surviving hypoxic cells would re-oxygenate, enter cycle and eventually provide a focus for tumour regrowth.

There is much evidence, however, that reoxygenation can occur *during* a course of treatment which reduces the numbers of hypoxic cells and is therefore beneficial. This is one of the contributory factors to the superior efficacy of multi-fraction treatments. However, it is the hypoxic cells that remain after treatment is completed that are a source of tumour regrowth. Most solid tumours, human as well as experimental, exhibit some necrosis quite early in their development and even in apparently well-vascularised

© The Macmillan Press Ltd., 1984.

regions of tumours, micro necrotic foci are often observed in histological preparations. There is no doubt that hypoxia is the major influence on radiation response of most experimental solid tumours treated with single doses of radiation. There is equally no doubt, however, that in these experimental systems, re-oxygenation during *fractionated* treatment reduces, and in some cases eliminates, the burden of hypoxic cell resistance. Re-oxygenation must be an important factor in the response of human tumours to radiation. The vexing question though remains: To what extent does re-oxygenation reduce the number of hypoxic cells and in which tumours is residual hypoxia a cause of treatment failure? Some clinical trials of radiotherapy of haemoglobin status (Hank & Smith, 1977; Bush *et al.*, 1978; Dische *et al.*, 1983) indicate that hypoxia certainly can be a factor in the response of some cancers of the cervix and the head and neck region. The problem, however, that bedevils such clinical trials is the means of identifying those tumours where hypoxia-cell resistance is an important factor in overall response and those where it is not. This problem is relevant, of course, to the current trials of chemical sensitizers for hypoxic cells.

*Chemical radiosensitizers*

Interest in the ubiquity of the oxygen effect that developed in the late fifties and early sixties prompted the development of chemical agents that would "mimic" the oxygen effect. There are now many examples of such agents showing a wide diversity of chemical structure. With very few exceptions, sensitization occurs only in hypoxia and this remains the main rationale for differential radiosensitization, i.e. enhanced tumour response without a concomitant increase in normal tissue radiation morbidity.

While most *in vitro* chemical radiosensitizers, for one reason or another, show little if any sensitization *in vivo*, the nitroheterocycles are an exception, particularly the *nitroimidazoles*. Many compounds of this class show good tumour penetration due mainly to their favourable pharmacokinetic properties but their toxicological properties vary widely.

The drug Metronidazole or "flagyl" was investigated clinically as a radiosensitizer in the early seventies. While the drug is a modest sensitizer on a weight-for-weight basis, it was the first hypoxic cell sensitizer shown to be effective in experimental solid tumours. However, clinical studies soon showed that maximum tolerated doses were insufficient for substantial radiosensitization in human tumours.

By this time, it was well-established that the efficacy of radiosensitizers of this type was directly related to the oxidative or "electron-affinic" properties of the compounds. This led to the proposal that structural modifications of the nitroimidazole nucleus involving the transposition of the nitro group from the 5- to the 2-position in the ring should greatly increase the potency. This was confirmed for a range of such compounds. One drug, misonidazole showed much promise in that in almost all experimental tumour systems, large sensitization factors were observed.

Preliminary clinical studies showed good tumour penetration accompanied by some evidence of radiosensitization and randomized clinical trials were initiated, most of which are now complete. Overall, however, the results have been disappointing (collected papers). Evidence of early benefit was noted in some of these trials but much of this has been lost during extensive follow-up. The major problem has proved to be the neurological complications encountered with this drug which have prevented its use at dosages sufficient to give large sensitization factors. It may well be that many of the misonidazole trials so far concluded have been too small to show significant benefits if only a fraction of the patients involved had tumours where residual hypoxia was a cause of treatment failure.

In specialised circumstances, the problem of insufficient dosage has been overcome by ingenious techniques for local administration (collected papers) and these studies are yielding encouraging results. Further, recent analysis of results from an MRC trial of hyperbaric oxygen in the radiotherapy of advanced carcinoma of the cervix has provided further evidence concerning the influence of chronic anaemia in the development and location of tumour hypoxia (Dische *et al.*, 1983). This may have an impact on the identification of patient sub-groups that would benefit in future trials of radiosensitizers.

*New sensitizers*

Recent developments of new sensitizing drugs have been influenced by several factors. The neurotoxic properties of sensitizers of the misonidazole type are directly related to the lipophilic properties of the compounds and this has partly guided the development of less toxic drugs.

Two drugs currently in clinical trial are the misonidazole analogue SR 2508 developed as a radiosensitizer at the Stanford Research Institute and the Roche Compound Ro 032-8799.

SR 2508 is less lipophilic than misonidazole and, as expected, has proved to be much less neurotoxic in experimental animals. Phase I and II studies are in progress in the USA (collected papers) and there is clear evidence of better tumour penetration and more favourable tumour/plasma ratios than are obtainable with misonidazole. Further, substantially higher doses can be administered without encountering neurological complications. SR 2508 may prove to be near the optimum compound with respect to the lipophilicity relationship. Although the fall-off of penetration with decreasing lipophilicity occurs in tumours at substantially lower values than for neural tissue, compounds with octanol-water partition coefficients not much lower than SR 2508 tend to show decreased efficiencies of tumour uptake (Brown & Workman, 1980).

The compound Ro 03-8799 also appears to be less toxic than misonidazole and somewhat more effective in experimental tumour systems. Usually, higher drug levels are found in these model tumours compared with those in normal tissues and results from Phase I clinical trials now in progress show that this is the case in human tumours also (collected papers). The lipophilicity of this drug is highly pH-dependent due to the acid-base characteristics of the side chain substituent. The higher acidity of the extracellular fluid in hypoxic tissues may be a factor responsible therefore for the more favourable tumour uptake.

The search for new and more potent sensitizers is underpinned, as it must be, by the study of mechanisms. Oxygen itself acts as a radiation modifier by two distinct free radical mechanisms and there is evidence that is also true for sensitizers of the misonidazole type. Recently, however, mechanistic studies have tended to concentrate on methods of "biochemical manipulation" of the cell.

The compound buthionine sulphoximine (BSO) is an inhibitor of glutathione synthesis in mammalian cells due to its suppression of the $\gamma$ glutamyl cysteine synthetase step in biosynthesis chain. Sensitization due to misonidazole or its analogues is enhanced when this inhibitor is administered prior to irradiation either *in vitro* or *in vivo*. Similar enhanced sensitization can also be achieved by the administration of agents which react directly with intracellular glutathione.

The origin of this enhanced sensitization almost certainly involves the inhibition of intracellular repair processes. Such inhibition could be exploited in a clinical sense provided there was a basis for a true differential effect. Repair inhibition in tumour cells is demonstrable and it is encouraging that the enhanced sensitization efficiency of electron-affinic sensitizers by SH-suppressive agents appears to be dependent to some extent on reduced oxygenation. It is too early though to speculate whether or not this approach will have any clinical application.

Some promise for the development of more potent sensitizers lies in observations that some compounds of the electron-affinic type are much more efficient than would be predicted from their redox properties. Examples of these are the "mixed function" compounds. The sensitizer RSU 1069 and its derivatives are nitroimidazoles containing a monofunctional alkylating group in the side chain. Some of these compounds are more efficient than misonidazole by at least an order of magnitude both *in vitro* and *in vivo* (Adams *et al.*, 1984). A contributory factor in this is the DNA reactivity which may be a mechanism of localisation of the sensitizer. It is well-established that enhancement of radiation damage in DNA is a major mechanism of sensitization by oxygen and other electron-affinic sensitizers.

### *Chemosensitization*

Evidence for the resistance of hypoxic tumour cells to radiation raises the question: Are hypoxic cells relatively resistant to the cytotoxic action of some anti-cancer drugs? The answer is probably yes. Hypoxic cells in tumours are located in poorly-vascularised regions and drug access may be limited. Further, oxygen is likely to be required for a cytotoxic response to some chemotherapeutic drugs particularly if energy-dependent processes are involved in the mechanisms of action. There are various reports that mammalian cells *in vitro* are less sensitive to the action of various anti-cancer drugs including some anthracyclines and anti-metabolites.

Some drugs of the nitroimidazole class are unusual in that they are much more cytotoxic under hypoxic conditions. Metronidazole, for example, is used routinely as an antibiotic for various anaerobic infections and other nitroimidazoles have found application in the treatment of some parasitic infections where there is an anaerobic vector in the mechanism. These agents are also differentially cytotoxic to hypoxic mammalian cells and this has prompted their investigation as anti-cancer agents aimed at attacking hypoxic cells in tumours.

It has been found that treatment of tumour-bearing mice with misonidazole, or other nitroimidazole analogues increases the response of these tumours to subsequent treatment with some other drugs – particularly alkylating agents and nitrosoureas. The increased anti-tumour response is *not* due, however, to the selective action of each drug against the respective oxic and hypoxic sub-populations but is rather a true potentiation phenomenon. Enhanced chemosensitivity is much greater in tumours than in normal tissue indicating a clear basis for increased therapeutic benefit.

Various mechanisms for chemosensitization have been proposed (collected papers) but the evidence is now compelling that a key step in the process involves a hypoxia-dependent process. The activity of nitroimidazoles used as anaerobic antibiotics rests on the reducibility of the nitro group. Anaerobic metabolic reduction of these compounds also occurs in mammalian cells and there is some support for the view that a reduced intermediate is involved in the overall chemosensitization process.

Whether or not chemosensitization will ultimately find a role in the clinic remains to be seen. However, the evidence that the cytotoxic activity of one drug can be enhanced by another in a process mediated through tumour hypoxia is encouraging since it provides a basis for a *differential* antitumour response.

MRC Radiobiology Unit, Harwell,

Didcot, Oxon OX11 0RD.

G.E. Adams

### References

ADAMS, G.E., AHMED, I., SHELDON, P.W. & STRATFORD, I.J. (1984). Radiation sensitization and chemopotentiation RSU 1069, a compound more efficient than misonidazole *in vitro* and *in vivo, Br. J. Cancer,* **49,** Suppl. V, 571.

BROWN, J.M. & WORKMAN, P. (1980). Partitition coefficient as a guide to the development of radiosensitizers which are less toxic than misonidazole. *Radiat. Res.,* **82,** 171.

BUSH, R.S., JENKIN, R.D.T., ALLT, W.E.C. & 4 others. (1978). Definitive evidence for hypoxic cells influencing cure in cancer therapy. *Br. J. Cancer,* **37,** Suppl. III, 302.

COLLECTED PAPERS. (1984). In "Chemical Modifiers of Cancer Treatment". *Int. J. Radiat. Oncol. Biol and Phys.,* (in press).

DISCHE, S., ANDERSON, P.J., SEALEY, R. & WATSON, E.R. (1983). Carcinoma of the cervix – anaemia, radiotherapy and hyperbaric oxygen. *Br. J. Radiol.,* **56,** 251.

HENK, J.M. & SMITH, C.W. (1977). Radiotherapy and hyperbaric oxygen in head and neck cancer. *Lancet,* **ii,** 104.

SUIT, H.D. (1982). Potential for improving survival rates for the cancer patient by increasing the efficacy of treatment of the primary lesion. *Cancer,* **50,** 1227.

THOMLINSON, R.H. & GRAY, L.H. (1955). The histological structure of some human lung cancers and the possible implication for radiotherapy, *Br. J. Cancer,* **9,** 539.